# Relationships Between Quality of Life and Emotional Resilience for Those With and Without Acne

**DOI:** 10.1111/jocd.16744

**Published:** 2025-01-06

**Authors:** Caroline J. Stone, Nicole Ufkes, Aaron M. Secrest, Maureen A. Murtaugh, Megan E. Vanneman, Ashley M. Snyder

**Affiliations:** ^1^ School of Medicine University of Utah Salt Lake City Utah USA; ^2^ Department of Dermatology University of Utah Salt Lake City Utah USA; ^3^ Department of Population Health Sciences University of Utah Salt Lake City Utah USA; ^4^ Department of Dermatology Christchurch Hospital, Health New Zealand Te Whatu Ora Christchurch New Zealand; ^5^ Division of Epidemiology, Department of Internal Medicine University of Utah Salt Lake City Utah USA; ^6^ Department of Nutrition & Integrative Physiology University of Utah Salt Lake City Utah USA

**Keywords:** acne, mental health, quality of life, resilience

Although not life‐threatening or physically debilitating, acne vulgaris has a greater psychological impact than many other chronic conditions [1]. Compared with patients with asthma, epilepsy, back pain, arthritis, or psoriasis, patients with acne experience similar, if not greater, impairment in mental health scores [1]. Resilience helps maintain mental health in vulnerable populations undergoing stressors [2]. Despite extensive research on mental health and acne, there is a notable lack of data exploring the connection between skin‐related quality of life (SRQL) and emotional resilience in patients with acne.

Resilience, the ability to adapt to stress and adversity [2], is essential for maintaining mental health. Emotional resilience can mitigate the psychological impact of stressors, leading to better mental health outcomes and improved satisfaction with clinical care [2]. This exploratory study aimed to address how emotional resilience, measured using the Connor–Davidson Resilience Scale (CD‐RISC), correlates with SRQL, measured using the Skindex‐16, in patients with acne versus acne‐free controls.

The Skindex‐16 is a validated patient‐reported outcome measure that assesses the effects of skin disease on quality of life across three domains: physical symptoms like itching and irritation, emotional responses such as worry or embarrassment, and functional impacts including difficulties with social interactions and work [3]. We used data from a previously published project on acne and lifestyle among adolescents and young adults [4]. Individuals between the ages of 12 and 24 years, with and without acne were included. Controls were excluded if they ever used isotretinoin and/or if they had a chronic inflammatory skin disease flaring at their dermatology visit. For both cases and controls, female participants could not be pregnant or breastfeeding or ever have been diagnosed with polycystic ovary syndrome. Only participants with complete data were included, resulting in a sample of 195 participants, with an average age of 18.7 years and ranging from 12 to 24 years. Most participants were female (*n* = 120 [61.5%]) and non‐Hispanic White (*n* = 159 [81.5%]) (Table 1). Most participants had left secondary education and were attending college or trade school after high school (*n* = 76 [39.0%]), were attending graduate school (*n* = 11 [5.6%]), or had left school entirely (*n* = 27 [13.8%]). The rest were in middle school (*n* = 24 [12.3%]) or high school (*n* = 57 [29.2%]). Forty‐seven (24.1%) had full‐time employment, 68 (34.9%) worked part‐time jobs, and 2 (1.0%) had both full‐time and part‐time jobs. Among acne cases, severity ranged from almost clear to severe. Skindex‐16 instructions (but not questions) were modified to ask participants to reflect specifically on their acne or other skin condition.

**TABLE 1:** Descriptive statistics of demographics, emotional resilience scores, and quality of life scores by acne cases versus acne‐free controls.

	Case (*N* = 130)	Control (*N* = 65)	*p* ^a^
Age, mean ± SD	18.0 ± 3.4	20.0 ± 4.0	< 0.01
Sex, *n* (%)			
Female	82 (63.1)	38 (58.5)	0.53
Male	48 (36.9)	27 (41.5)	
Race/ethnicity, *n* (%)			
Non‐Hispanic White	99 (76.2)	60 (92.3)	< 0.01
Other	31 (23.8)	5 (7.7)	
Emotional resilience, mean ± SD	69.0 ± 14.9	71.9 ± 13.1	0.19
Skindex‐16 symptoms, mean ± SD	23.2 ± 20.0	7.4 ± 11.1	< 0.01
Skindex‐16 emotions, mean ± SD	46.5 ± 28.1	13.5 ± 21.9	< 0.01
Skindex‐16 functioning, mean ± SD	15.9 ± 21.0	5.1 ± 11.7	< 0.01

^a^ Student's *t*‐test or chi‐squared test.

Continuous and categorical variables were assessed using Student's *t*‐tests and chi‐squared tests, respectively. Spearman's rank correlation was used to assess the relationship between emotional resilience and SRQL variables. An *α* < 0.05 was considered statistically significant. Stata version 16.1 was used for analyses.

The mean (± standard deviation) emotional resilience score was 69.9 (± 14.4). Mean scores for Skindex‐16 symptoms, emotions, and functioning domains were 18.0 (± 19.0), 35.5 (± 30.5), and 12.3 (± 19.1), respectively. All three domains had negative correlations with emotional resilience: symptoms (*ρ* = −0.15, *p* = 0.04), emotions (*ρ* = −0.26, *p* < 0.01), and functioning (*ρ* = −0.29, *p* < 0.01), indicating that greater emotional resilience correlated with improved SRQL. When assessing values by acne cases versus acne‐free controls, emotions and functioning had significant negative correlations with emotional resilience among cases while symptoms had a significant negative correlation with emotional resilience among controls (Table 2). These findings suggest that while symptoms are significantly related to SRQL in acne‐free individuals, they do not have the same impact on those with acne. Instead, the emotions and functioning domains appear more relevant for patients with acne.

**TABLE 2:** Spearman's correlation coefficients illustrating the correlations emotional resilience has with Skindex‐16 domain scores by acne cases versus acne‐free controls.

	Cases (*N* = 130)		Controls (*N* = 65)	
	*ρ*	*p*	*ρ*	*p*
Symptoms	−0.04	0.64	−0.29	0.02
Emotions	−0.26	< 0.01	−0.19	0.13
Functioning	−0.30	< 0.01	−0.21	0.10

Our study aligns with existing literature indicating that lower emotional resilience is linked to poorer SRQL in patients with acne. In one study, over two‐thirds of adolescent participants and all adult participants reported experiencing impacts on social activities resulting from acne [1]. Further evidence indicates a strong association between psychological symptoms and low resilience in patients with acne, though the difference in resilience between patients with acne and controls was not significant [5]. However, their inclusion of an older cohort (ages 18 to 35) may help explain differences in results compared with the current study [5]. Our study included only adolescents and young adults between the ages of 12 to 24 years. Furthermore, because emotional resilience is an underexplored factor in relation to the mental and emotional health of patients with acne, we did not have significant evidence for power calculations, and so it is possible our sample was underpowered. We hope our work provides evidence for designing well‐powered studies in the future.

In conclusion, our results illustrate that emotional resilience is associated with better SRQL among patients with acne. Dermatologists and other healthcare providers should consider ways to enhance resilience to improve outcomes when patients struggle with cosmetic and other problems from acne. Further research is needed to evaluate the clinical benefits of using the CD‐RISC with acne patients and to develop targeted interventions that enhance resilience and SRQL in this population.

## Author Contributions

Dr. Snyder developed the initial idea for this study with assistance from Dr. Secrest. Ms. Stone, Dr. Secrest, Dr. Murtaugh, Dr. Vanneman, and Dr. Snyder were all involved in developing methods. Ms. Stone, Dr. Ufkes, Dr. Secrest, and Dr. Snyder were involved in recruitment/data collection. Dr. Snyder conducted the statistical analyses. Ms. Stone and Dr. Snyder wrote the initial draft, and all authors had a chance to edit a revised draft.

## Ethics Statement

This project received ethics approval from the University of Utah Institutional Review Board (#140574; approval received November 8, 2021). The participants in this manuscript gave electronic written informed consent.

## Conflicts of Interest

The authors disclose no conflicts of interest.

## Data Availability

The data underlying this article cannot be shared publicly due to privacy and ethical concerns because permission to share data was not given by participants.
